# An interactive application for malaria elimination transmission and costing in the Asia-Pacific

**DOI:** 10.12688/wellcomeopenres.14770.2

**Published:** 2019-07-29

**Authors:** Olivier J. Celhay, Sheetal Prakash Silal, Richard James Maude, Chris Erwin Gran Mercado, Rima Shretta, Lisa Jane White

**Affiliations:** 1 Mahidol Oxford Tropical Medicine Research Unit, Faculty of Tropical Medicine, Mahidol University, Bangkok, Thailand; 2 Modelling and Simulation Hub, Africa (MASHA), Department of Statistical Sciences, University of Cape Town, Rondebosch, Cape Town, 7700, South Africa; 3 Centre for Tropical Medicine and Global Health, Nuffield Department of Medicine, University of Oxford, Oxford, UK; 4 South African DST-NRF Centre of Excellence in Epidemiological Modelling and Analysis, Stellenbosch University, Cape Town, South Africa; 5 Harvard TH Chan School of Public Health, Harvard University, Boston, USA; 6 Department of Tropical Hygiene, Faculty of Tropical Medicine, Mahidol University, Bangkok, Thailand; 7 Global Health Group, University of California, San Francisco, 550 16th St, 3rd Floor, Box 1224, San Francisco, CA, 94158, USA; 8 Swiss Tropical and Public Health Institute, Socinstrasse 57, 4002 Basel, Switzerland; 9 University of Basel, Petersplatz 1, 4001 Basel, Switzerland

**Keywords:** Model-based Decision Support System, Interactive application, Malaria, Elimination, Modeling, Modelling, Asia-Pacific, GMS

## Abstract

Leaders in the Asia-Pacific have endorsed an ambitious target to eliminate malaria in the region by 2030. The emergence and spread of artemisinin drug resistance in the Greater Mekong Subregion makes elimination urgent and strategic for the global goal of malaria eradication. Mathematical modelling is a useful tool for assessing and comparing different elimination strategies and scenarios to inform policymakers. Mathematical models are especially relevant in this context because of the wide heterogeneity of regional, country and local settings, which means that different strategies are needed to eliminate malaria. However, models and their predictions can be seen as highly technical, limiting their use for decision making. Simplified applications of models are needed to allow policy makers to benefit from these valuable tools. This paper describes a method for communicating complex model results with a user-friendly and intuitive framework. Using open-source technologies, we designed and developed an interactive application to disseminate the modelling results for malaria elimination. The design was iteratively improved while the application was being piloted and extensively tested by a diverse range of researchers and decision makers. This application allows several target audiences to explore, navigate and visualise complex datasets and models generated in the context of malaria elimination. It allows widespread access, use of and interpretation of models, generated at great effort and expense as well as enabling them to remain relevant for a longer period of time. It has long been acknowledged that scientific results need to be repackaged for larger audiences. We demonstrate that modellers can include applications as part of the dissemination strategy of their findings. We highlight that there is a need for additional research in order to provide guidelines and direction for designing and developing effective applications for disseminating models.

## Abbreviations

APLMA, Asia Pacific Leaders Malaria Alliance

DSS, Decision Support System

GMS, Greater Mekong Subregion (Cambodia, Yunnan Province in China, Lao People’s Democratic Republic, Myanmar, Thailand and Viet Nam)

METCAP, Malaria Elimination Transmission and Costing in the Asia-Pacific

NMCP, National Malaria Control Programme

Pf, *Plasmodium falciparum*

Pv, *Plasmodium vivax*

WHO, World Health Organisation

## Introduction

Malaria cases and deaths in the Asia-Pacific have declined dramatically in recent decades. However, there is a marked rise in the occurrence of artemisinin resistance across the Greater Mekong Subregion (GMS), which threatens to reverse the gains made ^1^. As a response, leaders in the Asia Pacific region at the highest levels have endorsed a regional goal of making the Asia-Pacific malaria-free by 2030 and many countries across the region are now working towards national elimination of malaria ^2, 3^. Malaria Elimination Transmission and Costing in the Asia-Pacific (METCAP) is a cross-disciplinary project aimed at evaluating and comparing potential malaria control and elimination strategies for 22 countries in the Asia-Pacific region: Afghanistan, Bangladesh, Bhutan, Cambodia, DPR Korea, India, Indonesia, Lao PDR, Malaysia, Myanmar, Nepal, Pakistan, Papua New Guinea, People’s Republic of China, Philippines, Republic of Korea, Solomon Islands, Sri Lanka, Thailand, Timor-Leste, Vanuatu, Viet Nam. At the core of METCAP is a dynamic epidemiological-economic multi-patch model of the transmission and costing of malaria that project malaria incidence until 2030 across several scenarios (i.e. using several packages of interventions for the elimination of malaria).

The METCAP model is the first mixed *Plasmodium falciparum* ( *Pf*) / *Plasmodium vivax* ( *Pv*) mathematical model of its kind. The METCAP model was built in three stages. First, country-specific information were obtained from several sources, including WHO’s annual World Malaria Reports (2008; 2010–2015) ^4^, published literature on glucose-6-phosphate dehydrogenase deficiency (G6PDd) prevalence ^5^ and the Earth System Research Laboratory website for El Niño Southern Oscillation time series ^6^. This data was used to build ranges of plausible estimates of several malaria-related indicators, including annual disease burden estimates ^7^. The second stage consisted of modelling several indicators (such as the estimated incidence of all malaria species and reported fatalities) for each country between 2016 and 2030, under scenario-specific assumptions. A total of 80 scenarios were built based on 10 different sets of packages of interventions. These ranged from discontinuing most malaria control activities to a very substantial scale-up of interventions, which could be supplemented by mass drug administration (MDA) or an increase in insecticide-treated net coverage, meanwhile assuming different trajectories of drug resistance (increasing or stable) ^8^. The third and final stage was a full costing of each scenario that was done evaluating the costs of interventions per country, year and component ^9^. This multi-patch model was developed in R language ^10^ with calls to C++ routines to find numeric solutions to the model’s ordinary differential equations (ODE).

The applicability of models for decision-making has been questioned by policymakers: with reactions ranging from them believing them completely or mistrusting them completely. Clearly the right place is between the two ^11^. Communicating the results of models effectively so as to eliminate biases and allow policymakers themselves to arrive at wise judgements is a difficult goal to attain ^12^. Policymakers, especially in developing countries, often report difficulties with the format and style in which research outputs are presented, stating that research reports are often written in an academic style using technical language and include complex statistics that are difficult to understand. On the other hand, researchers may feel that oversimplifying research findings will omit relevant details needed to fully understand the research problem. Researchers may also be concerned about the academic rigor of their work requiring details of research methodology and the use of technical terminology ^13^. Decision support systems (DSS) are computer-based information systems that support decision-making activities by giving access to information organised to inform judgments and preferences about a range of intervention options and their trade-offs. Several DSS of different design have been developed and used for malaria control strategies. The objectives driving the development of these DSS include supporting the diagnosis of malaria ^14^, targeting activities for malaria elimination in Bhutan ^15^, providing access to global maps of malaria transmission ^16^ and offering a mathematical modelling platform for population level models of malaria elimination ^17^.

For clarity, hereafter we will refer to DSSs or interactive application as “apps”, and use “App” with a capital letter when referring to the METCAP application ^18^.

## Methods

### Development background

The METCAP project is a multi-disciplinary collaboration with a team comprised of data scientists, modellers, epidemiologists, health economists and other experts. Inputs from this diverse team were instrumental in designing an App to specifically account for the different capacities, preoccupations, perceptions, and needs of its intended users, as well as the characteristics of the institutions in which they are working ^19^.

For the diverse targeted audience, different capabilities and needs of users were taken into account and several design strategies meant to mitigate hindering factors were developed ( Table 1). The main challenge in developing such an App revolves around finding a good balance in the information load, so that the user can access information quickly and conveniently without being overwhelmed. During the App building process, we discussed the target audience for the App, and considered potential trade-offs between simplifying the presentation of results and providing enough relevant background on the model and methods. At the core of this discussion was the task of identifying which important features are most difficult to communicate to policy-makers.

**Table 1.** Potential limiting factors for App use and resolution strategies.

Factors determining the App understanding and use	Details	Strategies for addressing factors
Computer literacy and ease of navigating web interface	User cannot navigate efficiently through the App and becomes discouraged.	Develop the App in accordance with web design principles. Make the App openly accessible on the web.
English language literacy	App is used in Asia-Pacific where English is, in most countries, a secondary language	Limit the amount of text in the App.
Technical knowledge of malaria	Too frequent use of technical terms will force the user to do outside research and possibly lead to their discouragement.	Provide a glossary of malaria related terms, and as many reminders of the acronyms as possible. Provided contextual directions directly in the App and links to more detailed information.
Technical knowledge of modelling	The user thinks that use of the App is restricted to people with good knowledge of modelling.	Insert videos to introduce users to mathematical modelling. Provide information on the specific model being developed: overview of methodology, uncertainty associated with the model, etc. Show all steps of construction of the model.
Attitude/perception/curiosity towards modelling	Modelling is perceived as too abstract and remote from on the ground realities. There is no real perception of the advantages of using modelling.	Make the experience like a conversation by allowing users to use their knowledge (choosing scenarios that make sense from an operational viewpoint). Encourage users to spend more time on the App by making the App engaging (interactive map, choice of colours etc.).

### App design

Historical epidemiological estimates, mathematical models, and costing were developed in collaboration during the project but are distinct pieces of work ^7– 9^.

**Figure 1.**
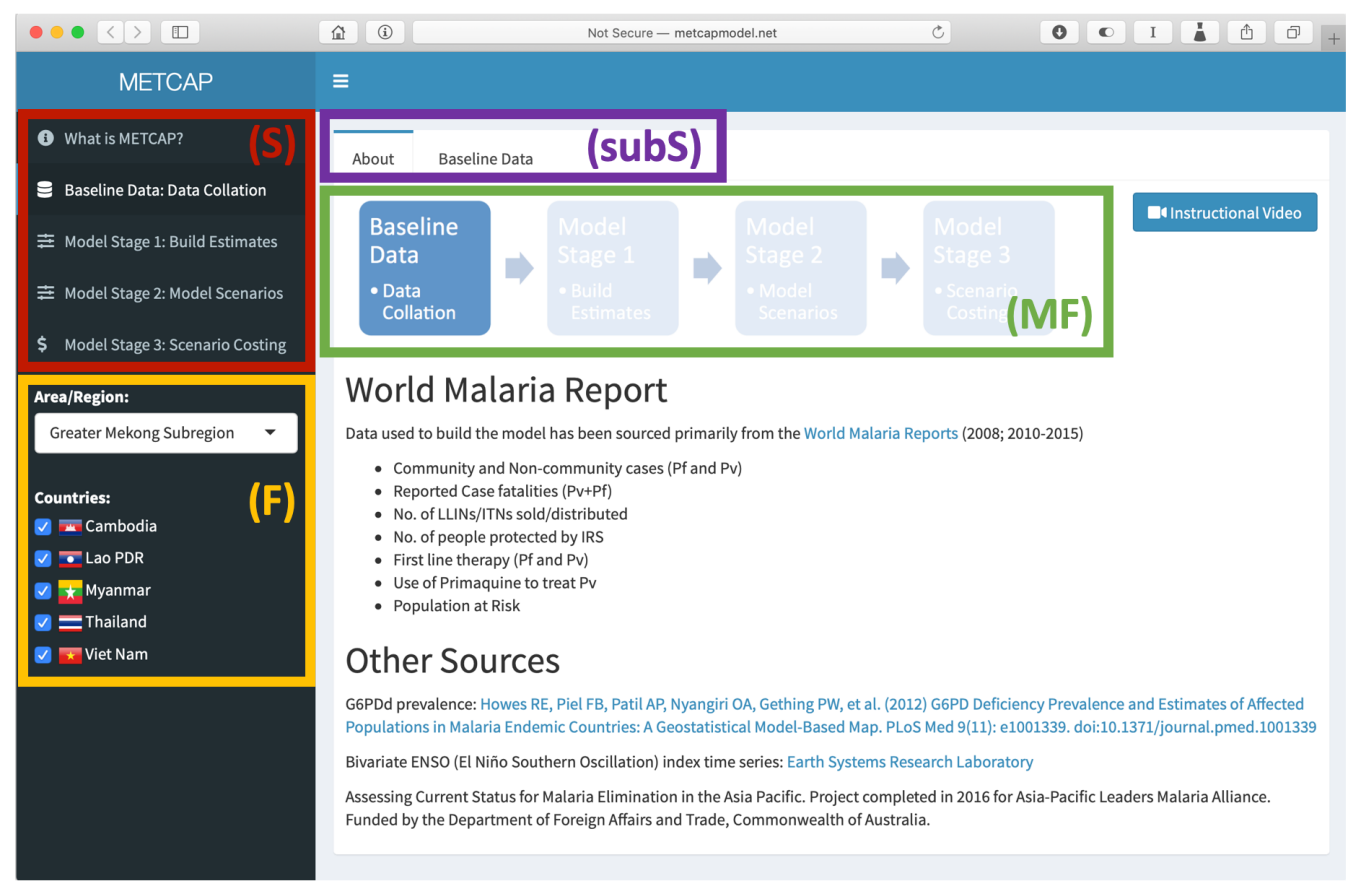
The App is made of five main sections (S) and each section contains several subsections (subS). All sections open with an “About” subsection that provides contextual information. In four of the five sections, the intended navigation is made explicit with the insertion of a diagram representing the menu flow (MF) and highlighting what stage of development of the model the user is looking at. These four sections also contain a panel (F) that allow the selection of an Area/Region or of one or several countries.

When considering trade-offs for the user interface (UI) ( Figure 1), the usability of the tool remained the first consideration. The visual attractiveness (look and feel) of the App was also deemed important. During informal internal discussions, ideas were shared and the App was incrementally updated. For example, the first prototype of the App did not contain any maps as we were concerned that the large surface areas of countries could lead to misrepresentation. We determined that the risk was mitigated by the presence of a grid plot with an equal size for every country below most maps. In one subsequent version of the App, we added the possibility to select several groups of countries that were grouped based on the composition of WHO regional offices (e.g. countries that are part of the WHO Regional Office for South-East Asia).

The mathematical model requires a significant computation time to run (from 30 minutes to two hours depending on the server) as well as a validation of the results by the expert modeler. Allowing the user to create their own package by adding different interventions by running the model live would not have been practical. To create the semblance of a live run, we simulated all possible combinations of interventions in advance, to be referenced by a series of check boxes. While testing prototypes of the App, these packages of interventions were frequently refined and added, leading to a more complete final App.

### Target audience’s relevant interests

The target audience for the App is comprised of decision makers at international, regional and national levels with an interest in the control or elimination of malaria in one area or across the whole Asia-Pacific region. This broad target audience can be disaggregated into several more specific groups according to their technical background and previous exposure to the subject of modelling and their main interests relevant to the METCAP project. We define four broad categories of users and summarise what we project as their main interests in the App in Table 2. (One clear limitation of this method is that the heterogeneity within a category can be very significant.)

**Table 2.** Anticipated main interests for the four identified audience groups.

Target audience	Anticipated main interests
Donors and high-level policymakers	• Optimal long-term strategy for malaria elimination. • Limitations and uncertainty associated with the model. • Associated costs for the “minimal elimination scenario”.
Senior staff from National Malaria Control Programs (NMCPs) of the 22 countries	• Feasibility of malaria elimination for a specific country. • Optimal scenarios for elimination in a specific country with respect to country strategy (e.g. acceptability of mass drug administration). • Global costs for each scenario.
Technical health agencies (e.g. World Health Organisation) staff	• Quality of underlying surveillance data. • Indicators chosen and their relation to elimination. • Strategies for drug resistance containment.
Modellers and other researchers	• Underlying data and assumptions made to build the model. • Modelling methods used. • Model results.

Before and during the development of a prototype of the App, the development team defined and reframed its objectives and strategies for reaching this audience. We identified four main objectives:

(1) Engage with a diverse target audience and provide them with an accessible, engaging, not misguiding and technically sound presentation of the METCAP model results and underlying data. This presentation should match the audience’s interests and provide enough contextual information to allow them to form a sound, balanced, judgment of the data provided.
(2) Display a large amount of information in a cohesive way through an intuitive interface that maximises accessibility for a variety of audiences. At the same time, provide contextual information on the underlying data (source, quality, uncertainty, etc.) and the methods.
(3) Provide information on the mathematical model used along with a more general presentation on the topic of modelling and compartmental models. Make explicit the assumptions made and the limitations of the work by displaying the sequence that led to developing the model and by highlighting the potential limitations of the model.
(4) Allow user to delve into specific contexts according to their interests, such as a specific country or group of countries, a specific set of scenarios or specific indicators. For example, the potential impact on *Pf* incidence of mass drug administration in the GMS.

### Implementation

The model was run using R Statistical Software ^10^, with calls to scripts in the C++ language and the results were stored and saved in R objects. We used R Statistical Software and extended it with the R Shiny package v1.1.0 ^20^, an extension that is a “web application framework” combining R as a backend server and a classic HTML User Interface. Shiny allows for customisation of the App’s UI to provide an elegant environment for displaying user input controls and simulation output, where the latter simultaneously updates with changing input ^21^. Several solutions for developing interactive apps exist but we decided on Shiny for its convenience. Shiny is an open-source, free, package of R—the same language ecosystem that was used in the modelling section.

### Operation

The App ^18^ can be publicly accessed at http://www.metcapmodel.net with a recent version of a modern browser (e.g. Firefox, Google Chrome, Safari, Internet Explorer). The App is most responsive when accessed from an internet connection with a download speed of at least 1 megabit per second (Mbps).

## Use cases

With the objective of demonstrating to policy-makers that malaria elimination in the Asia-Pacific is a sensible investment, researchers from the Global Health Group at the University of California, San Francisco (UCSF) used to the outputs from the App to develop the economic rationale of malaria elimination in several national and regional settings. This led to the development of five "elimination investment cases”; reports investigating the costs and economic benefits of malaria elimination in the Asia Pacific Region, Greater Mekong Subregion, Bangladesh, Indonesia and in Papua New Guinea ^22^.

Below, we describe the different parts of the App while highlighting which elements were instrumental in the development of one of these five reports: *An Investment Case for Eliminating Malaria in the Greater Mekong Subregion (GMS)* (referred to subsequently as “investment case paper”) ^23^.

Upon the initial rendering of the App, a modal window opens with a disclaimer that informs the viewer of the limitations of the data provided. After this disclaimer, the user is able to access the first of five sections, “What is METCAP?”, that contains contextual information on the project and an introduction to mathematical modelling. 

The section “Baseline Data: Data Collation” consist of an interactive choropleth map and several time series. The map of the selected area/region is colored by country based on an indicator to be selected in a dropdown menu (e.g. percentage of ITN coverage in 2014). Below the map, two plots show the year by year evolution from 2000 to 2015 for the indicator selected in a dropdown menu. One plot shows the value of the indicator aggregated to the area / region selected and below it appears a grid of plots with the indicator for each country. To generate the investment case paper figure *Figure 1 Confirmed cases and deaths of malaria in the GMS countries, 2000–2015*, one would select “Greater Mekong Subregion” in the left panel and “Total Cases” and “Reported Cases Facilities” in the dropdown menu.

**Figure 2.**
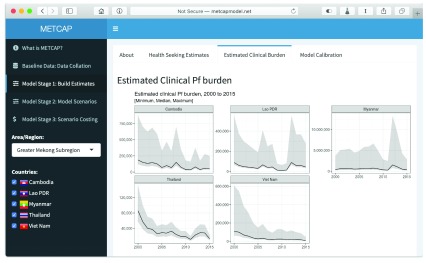
Grid plot of estimated clinical Pf burden for selected countries.

Central to the section “Model Stage 1: Build estimates” is the “Health Seeking Estimates” that displays a map colored with a sequential palette based on values of health seeking estimates. The next subsection displays the estimated clinical Pf/Pv burden by country with confidence intervals that are built upon the uncertainty of these health seeking estimates ( Figure 2). The Model Calibration subsection displays the superposition of the estimated clinical burden with the model predictions. While the investment case paper presents the epidemiological and cost predictions (see below), only the App provides a comprehensive overview of the input data used. 

**Figure 3.**
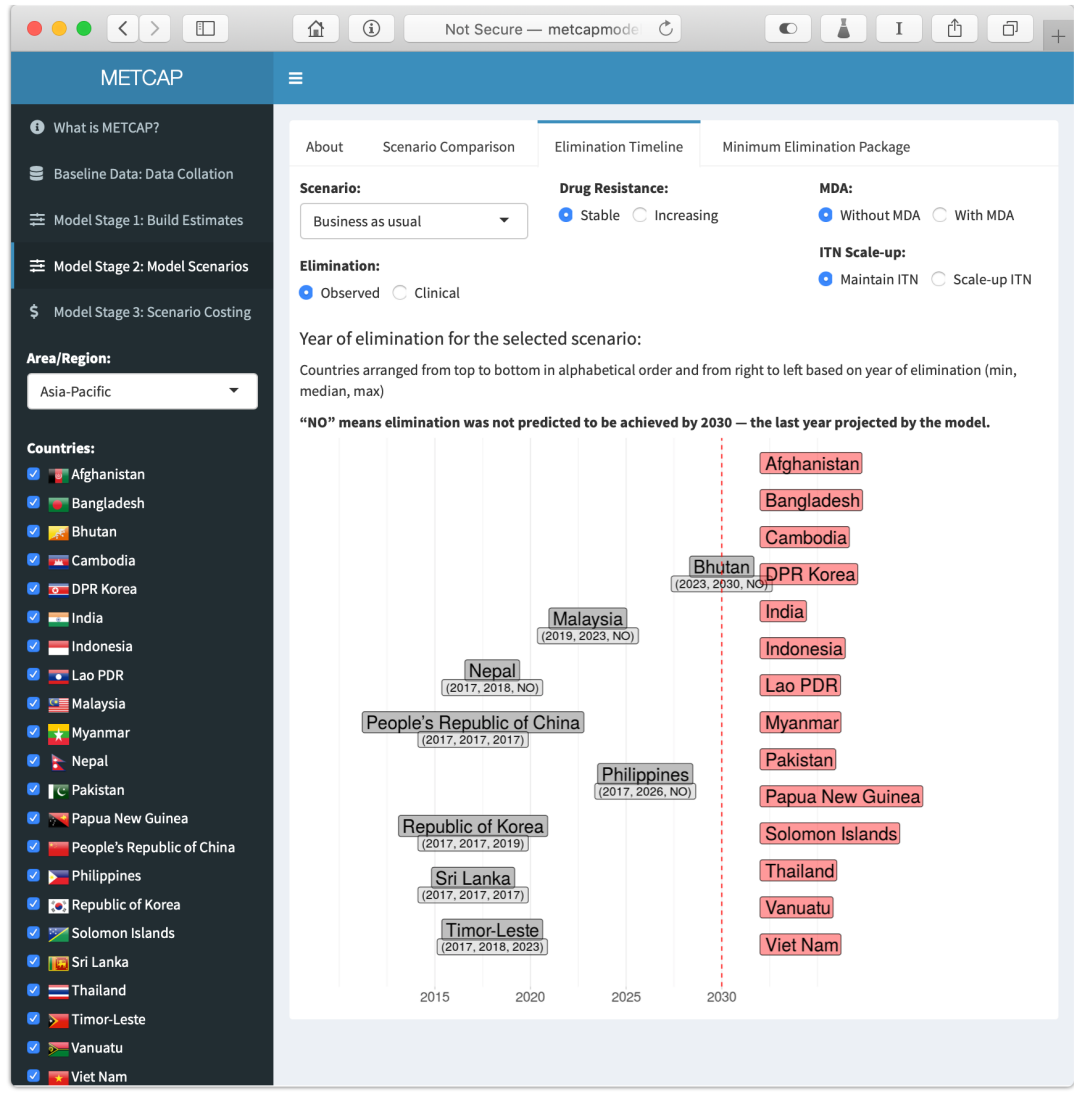
Timeline of predicted observed elimination year by the model under the “Business as usual” scenario without MDA while maintaining ITN coverage and assuming stable drug resistance.

The section “Model Stage 2: Model Scenarios” also offers several subsections to explore. “About” provides an explanation of the 10 different scenarios (package of interventions) and three kinds of assumptions (Stable/Increasing Drug Resistance, use of MDA and ITN Scale-up) for which numerical simulations can be displayed by the App. Further down the page appears an infographic of these 10 scenarios grouped into four categories (Reverse, Continue, Accelerate, Innovate) which is used in the investment case paper *Figure 7. Scenarios used in the transmission model.* The subsection “Scenario Comparison” allows the user to explore modeling results for one or several scenarios and different assumptions. The user can generate several individual country transmission plots exploring results that are tailored to a country context. For example, *Business as usual with Single dose new treatment* in Thailand and *New Pf drug with Mass Drug Administration and a scale up of ITN* in Cambodia. Some of these plots can be found in the investment case paper’s *Annex 3*. The subsection “Elimination Timeline” ( Figure 3) allows the user to select a scenario and to see the predicted year of elimination — or no elimination if the elimination is not predicted to be achieved by 2030. This graph was used to generate the investment case paper *Figure 9 Transmission prediction for GMS (2016-2030)*. The next subsection, *Minimum Elimination Package,* displays a map coloured depending on the minimum elimination scenario. That map was used in the investment case paper *Figure 8. Predicted minimum elimination scenarios*.

**Figure 4.**
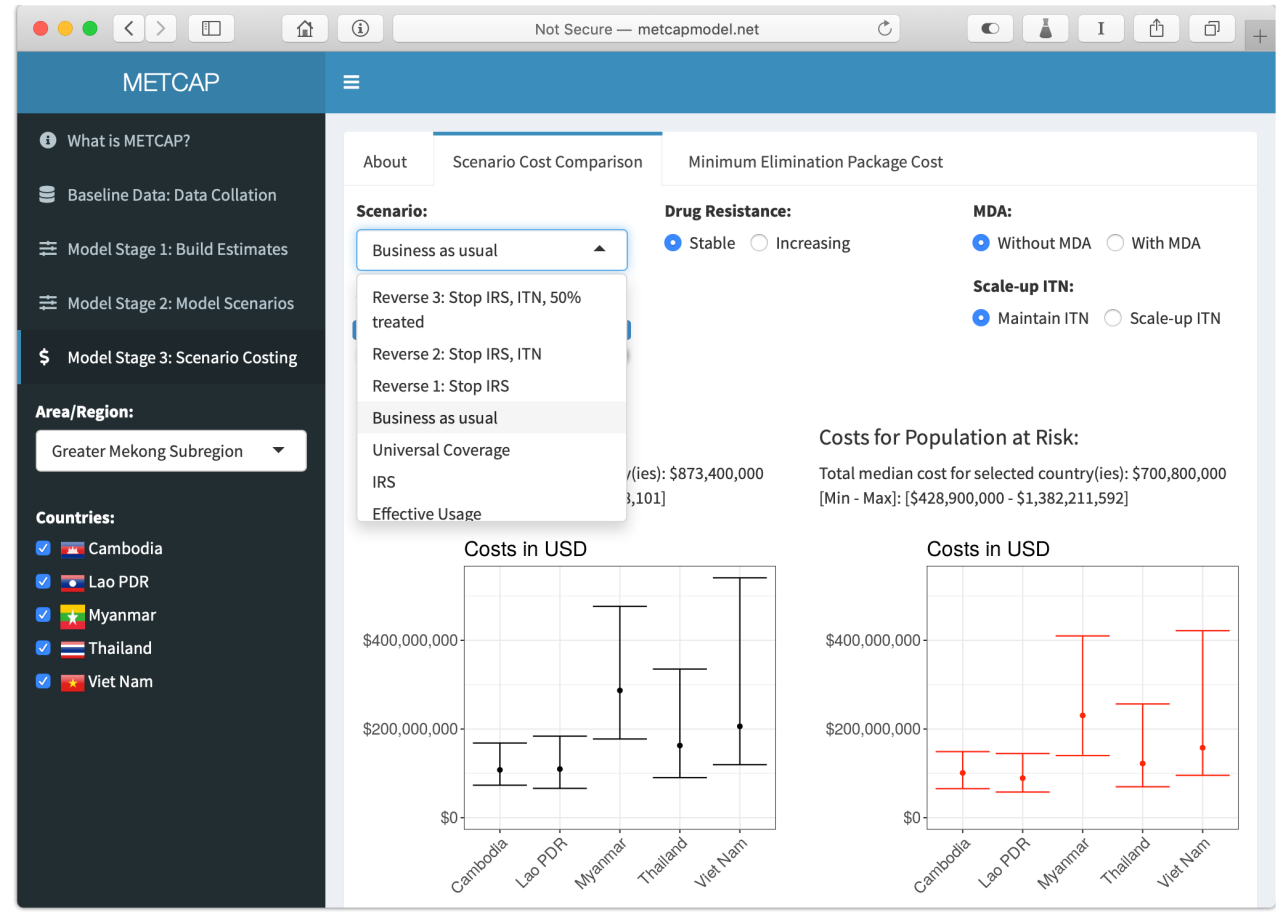
Total costs of malaria programs from 2016 – 2030 per country in the GMS in the scenario “Business as usual”.

In the last section, “Model Stage 3: Scenario Costing”, the subsection “Scenario Cost Comparison” displays the cost per country of a selected scenario ( Figure 4) as well as the area/region global cost per year. The subsection “Minimal Elimination Package cost” provides the cost for the scenario offering the minimal effort in each country as per the investment case paper *Table 6. Summary of costs and benefits. Minimum Elimination Package Cost*.

## Discussion

We created an App that provides users with convenient access to the results of modelling malaria elimination in the Asia-Pacific ^18^. The App is developed with open source technologies and could later be updated with new models and adapted to other settings (e.g. other regions, new indicators). The App is stable and easy to access through a web browser. Alongside the modelling results, we tried to show limitations related to uncertainty in the provided data and the nature of modelling. A more systematic analysis of user perceptions of the models could be achieved through observation techniques, task analysis, and other feedback methodologies. This would provide useful evidence for assessing which design Apps are better at communicating key messages and pitfalls.

All research in public health is done with the objective that the knowledge gained will somehow be used to improve health outcomes, and knowledge dissemination is recognised as an important component of the research process. The publication of scientific results in peer-reviewed journals is the unavoidable metric that dominates most researchers’ investment of resources in communicating their results. In order to make scientific articles more useful to policy makers, several journals have publication guidelines that request the inclusion of short sections to put research in context (e.g. Authors should state the implications for practice or policy of all research papers submitted to any journal in the Lancet family ^24^). There is a wide range in the types of papers which have considerable use to informing policy ^11^ but there are also practical limits to the amount of data that can be included and conveniently communicated within a paper following the pervasive format of an academic publication. Even if most papers include figures or tables that support data visualisation, they are typically static and, by design, do not allow the reader to interactively explore them. The format of the academic publication is especially limiting to the field of modelling where the amount of data used, the degree of data uncertainty, the voluminous complexity and variability of results and the range of questions that practitioners may need to apply to test the assumptions of the model or explore dimensions of the results can be considerable. If summarising is essential, providing direct access to data is required in such circumstances. Perceptions of the nature, uses and quality criteria of mathematical modelling in epidemiology are contradictory, even among the community of published authors in this field ^25^. The development of this discipline merits a framework for providing recommendations and guidance at various steps in the process, from design to reporting ^24^. It is vital that researchers pursue improvements in how they prepare and report research for the end users. The ability to communicate data, findings, and reports in commonly used language will aid decision makers in using all available evidence and tools for decision-making ^26^.

The recent implementations of free, open-source extensions for the most popular software platforms for data science (e.g. R, Python) allow the development of graphical user interfaces and web apps within these platforms (e.g. Shiny package for R ^20^, Dash framework for Python ^27^). The development of these extensions allows modellers to develop basic apps by themselves without requiring the support of expert software designers. We think that apps can offer a tremendous contribution by helping to build more relevant models for policymakers and supporting the communication of results. The design and development of dissemination tools concurrent with conducting modelling work means that policymaking questions are integrated in the early stages of model development and encourages considering and designing around what is most relevant to target audiences. An app can be provided alongside an article (as supplementary material) to be explored by the policy-maker on their own. Furthermore, this format of presentation could enhance the communication of concepts and data during interpersonal communications where the modeller, the policymaker and program level decision maker can interactively explore different options. The malaria ERAdication (malERA) group—the authoritative consultative group on modelling for malaria—has identified the development of interactive apps for models as a priority area for research in statistical modelling ^28^.

In order to promote the use of models more effectively, modellers must understand the needs of policymakers and be able to explain how modelling can support the decision-making process. Modellers must also be able to inform various audiences of the uncertainty of a model’s results, explain why a complex model is not necessarily superior to a simpler model, and generally help users navigate the added values and limitations of particular models. In addition to the generally challenging nature of communicating the models that we describe here, the sophistication of the METCAP model, the structural uncertainty of the underlying surveillance data and the high stakes of related decision-making (deciding between costly strategies), add a new dimension to the challenges in communication. We designed and developed the App with the goal of offering convenient access for an audience that might not otherwise have the time, resources or inclination to explore the METCAP project data and modelled results. Producing a cohesive, effective interface for an app is not a trivial task. To develop apps that are highly effective and have the maximum contribution to evidence-based policy-making, it is important to understand what factors have the potential to maximise an app utility and usefulness for end-users. We could not find any general guidance related to the design, development or dissemination of apps for public health. The rapid pace of development of technologies may explain why research is nascent in this area. Insights into the process of dissemination of apps are also of importance since they are relatively new in the field of public health and are thus not yet widely used, thus their adoption can be analysed through existing diffusion of innovations frameworks ^29^. Several techniques could be used to evaluate apps UI such as heuristic evaluation, usability testing, guidelines or cognitive walkthroughs ^30^. Since apps have a critical impact on the process-oriented aspects of decision-making, a combination of both outcome- and process-oriented evaluation measures is highly important for apps evaluation ^31, 32^. This is another area where research could be developed to help better understand what evaluation measures are the most relevant and could be applied.

Apps may be misleading through being excessively complex, poorly constructed, or not providing sufficient background information. This also strongly motivates a systematic evaluation of their potential use.

After a first prototype of the App was developed, several presentations of the project and the App were made to external audiences during informal meetings including Asian Development Bank, World Health Organisation, Global Fund, APLMA and National Malaria Control Programmes. There was no formal plan for dissemination of the App but rather an informal dissemination was executed on a needs basis as well as purposefully through networks of partners involved in malaria control and elimination activities in the region. Given that the App was part of a larger project to develop the evidence base for resource mobilization for elimination in the Asia Pacific region, the dissemination of app followed the plan for dissemination of the entire package of evidence. Opportunities for dissemination were identified in collaboration with APLMA and APMEN. These included several meetings during "malaria week" in Bangkok in 2017, APMEN annual and working group meetings in 2017 and 2018, partner meetings (for example, Regional Artemisinin Initiative steering committee meetings), WHO meetings and the World Malaria Congress in July 2018. The APMEN meetings included country level malaria programs as well as partner institutions.

The App server provides application usage metrics in the form of hours of use or number of connections in the past three months. Unfortunately, these metrics are not specific enough to form an accurate picture of the App usage. Integrating a user tracking script (e.g. Google Analytics) to the App would have been useful to gain insights on the number of connections by geographical area and origin of the visit (direct access, link in an email, link from a website, etc.), however, this was not done at the inception as the main objective of the project was to develop a product that would allow country level users to a rapid display of results in settings with slow internet. At the time, we did not want to risk slowing the App down by including additional scripts to track users. The priority of the App when developed was to provide policy level utility rather than having a pure research and evaluation utility. The authors recognize that this is a limitation of the project, however, this is being rectified in other app development projects.

Another limitation of the App is that only the preexisting scenarios can be selected and users cannot build packages by tailoring interventions beyond the options presented. To offer more flexibility to users, a similar country-level modeling app prototype is in development. This application allows the user to run a simplified version of the model live with greater flexibility in customising packages of interventions. With expected easier access to country-level users, plans are in place to quantify usability with more rigor.

## Conclusions

An interactive app for the exploration of mathematical models is an effective dissemination tool and may help to bridge the gap between evidence generation through modelling and policymaking. These are not intended to replace but rather to accompany peer-reviewed publications and to present scientific findings more effectively to policymakers. We have demonstrated that it is possible to develop an app that provides a substantial amount of data from the model in formats more accessible and useful to the typical decision maker. At every stage in the METCAP App’s development, the diverse audience of users was prioritised. We emphasize that with additional user research, we could develop more effective apps and encourage a multidisciplinary effort to a more systematic use of mathematical models.

## Data availability

All data underlying the results are available as part of the article and no additional source data are required.

## Software availability

The domain name metcapmodel.net is reserved until Jul 17 2027 and could be renewed to extend availability past this date. The App is hosted on the shinyapps.io server using Mahidol Oxford Research Unit (MORU)’s institutional shinyapps.io account. We do not anticipate any need for maintenance and it is expected that the App will remain available until mid 2027 at the metcapmodel.net address and for the foreseeable future at the address moru.shinyapps.io/METCAP. We could provide an offline version to users with poor or no internet connection and expect to be able to mobilise resources to provide maintenance or support if requested.

Software available from: www.metcapmodel.net.

Source code available at: https://github.com/ocelhay/METCAP/.

Archived source code at time of publication: https://doi.org/10.5281/zenodo.2437908 ^18^.

License: GNU General Public License, version 3.

## References

[1] Ashley EA Dhorda M Fairhurst RM: Spread of artemisinin resistance in *Plasmodium falciparum* malaria. *N Engl J Med.* 2014;371(5):411–423. 10.1056/NEJMoa1314981 25075834 PMC4143591

[2] APLMA Roadmap. Reference Source

[3] WHO Malaria Policy Advisory Committee and Secretariat: Malaria Policy Advisory Committee to the WHO: conclusions and recommendations of sixth biannual meeting (September 2014). *Malar J.* 2015;14(1):107. 10.1186/s12936-015-0623-5 25881119 PMC4404229

[4] WHO World Malaria Reports. Reference Source

[5] Howes RE Piel FB Patil AP: G6PD deficiency prevalence and estimates of affected populations in malaria endemic countries: a geostatistical model-based map. Seidlein von L, ed. *PLoS Med.* 2012;9(11):e1001339. 10.1371/journal.pmed.1001339 23152723 PMC3496665

[6] NOAA Earth System Research Laboratory: El Niño Southern Oscillation (ENSO). Reference Source

[7] Maude RJ Mercado CEG Rowley J: Estimating malaria disease burden in the Asia-Pacific [version 1; peer review: awaiting peer review]. *Wellcome Open Res.* 2019;4:59 10.12688/wellcomeopenres.15164.1

[8] Silal SP Shretta R Celhay OJ: Malaria elimination transmission and costing in the Asia-Pacific: a multi-species dynamic transmission model [version 1; peer review: awaiting peer review]. *Wellcome Open Res.* 2019;4:62 10.12688/wellcomeopenres.14771.1

[9] Shretta R Silal SP Celhay OJ: Malaria elimination transmission and costing in the Asia-Pacific: Developing an investment case [version 1; peer review: awaiting peer review]. *Wellcome Open Res.* 2019;4:60 10.12688/wellcomeopenres.14769.1 PMC6974926 32025571

[10] R Core Team: R: A Language and Environment for Statistical Computing. R Foundation for Statistical Computing, Vienna, Austria.2018 Reference Source

[11] Whitty CJ: What makes an academic paper useful for health policy? *BMC Med.* 2015;13(1):301. 10.1186/s12916-015-0544-8 26675206 PMC4682263

[12] Morss RE Demuth JL Lazo JK: Communicating Uncertainty in Weather Forecasts: A Survey of the U.S. Public. *Wea Forecasting.* 2008;23(5):974–991. 10.1175/2008WAF2007088.1

[13] Hennink M Stephenson R: Using research to inform health policy: barriers and strategies in developing countries. *J Health Commun.* 2005;10(2):163–180. 10.1080/10810730590915128 15804906

[14] Uzoka FME Osuji J Obot O: Clinical decision support system (DSS) in the diagnosis of malaria: A case comparison of two soft computing methodologies. *Expert Syst Appl.* 2011;38(3):1537–1553. 10.1016/j.eswa.2010.07.068

[15] Wangdi K Banwell C Gatton ML: Development and evaluation of a spatial decision support system for malaria elimination in Bhutan. *Malar J.* 2016;15:180. 10.1186/s12936-016-1235-4 27004465 PMC4804570

[16] Hay SI Snow RW: The malaria Atlas Project: developing global maps of malaria risk. *PLoS Med.* 2006;3(12):e473. 10.1371/journal.pmed.0030473 17147467 PMC1762059

[17] Maude RJ Saralamba S Lewis A: Modelling malaria elimination on the internet. *Malar J.* 2011;10: 191. 10.1186/1475-2875-10-191 21756319 PMC3160427

[18] Source code of the App: ocelhay/METCAP: Manuscript release (Version v2.0). *Zenodo.* 2018 10.5281/zenodo.2437908

[19] Moss RH: Assessing decision support systems and levels of confidence to narrow the climate information “usability gap”. *Clim Change.* 2016;135(1):143–155. 10.1007/s10584-015-1549-1

[20] Chang W Cheng J Allaire J: shiny: Web Application Framework for R. http://shiny.rstudio.com.2017; Accessed September 22, 2016. Reference Source

[21] Wojciechowski J Hopkins AM Upton RN: Interactive Pharmacometric Applications Using R and the Shiny Package. *CPT Pharmacometrics Syst Pharmacol.* 2015;4(3):e00021. 10.1002/psp4.21 26225240 PMC4394611

[22] UCSF Global Health Group’s Malaria Elimination Initiative (MEI): Financing Elimination in the Asia Pacific. Reference Source

[23] UCSF Global Health Group’s Malaria Elimination Initiative (MEI): Elimination Investment Cases. Reference Source

[24] The Lancet Information for Authors. Reference Source

[25] Hejblum G Setbon M Temime L: Modelers' perception of mathematical modeling in epidemiology: a web-based survey. Scalas E, ed. *PLoS One.* 2011;6(1):e16531. 10.1371/journal.pone.0016531 21304976 PMC3031574

[26] McCaughey D Bruning NS: Rationality versus reality: the challenges of evidence-based decision making for health policy makers. *Implement Sci.* 2010;5:39. 10.1186/1748-5908-5-39 20504357 PMC2885987

[27] Dash, an Open Source Python library for creating reactive, Web-based applications. Reference Source

[28] malERA Consultative Group on Modeling: A research agenda for malaria eradication: modeling. *PLoS Med.* 2011;8(1):e1000403. 10.1371/journal.pmed.1000403 21283605 PMC3026697

[29] Rogers EM: Diffusion of Innovations.5 ed. Reference Source

[30] Jeffries R Miller JR Wharton C: User interface evaluation in the real world: a comparison.1997;1–6. Reference Source

[31] Phillips-Wren GE Hahn ED Forgionne GA: A multiple-criteria framework for evaluation of decision support systems. *Omega.* 2004;32(4):323–332. 10.1016/j.omega.2004.01.003

[32] Forgionne GA: An AHP model of DSS effectiveness. *Eur J Inf Syst.* 1999;8(2):95–106. 10.1057/palgrave.ejis.3000322

