# Supplementary material for: An interactive application for malaria elimination transmission and costing in the Asia-Pacific
Source: Wellcome Open Res. 2019 Jul 29;4:61. Originally published 2019 Apr 1. [Version 2] doi: 10.12688/wellcomeopenres.14770.2 (PMC6971843; doi:10.12688/wellcomeopenres.14770.2)
Supplement: Peer review history

# Peer review: Reviewer response for version 2

**DOI:** 10.21956/wellcomeopenres.16763.r37542

**Published:** 2020-01-20

**Authors:** Maxine Anne Whittaker

**Affiliations:** 1 College of Public Health, Medical and Veterinary Sciences, James Cook University, Townsville, Australia

- The authors have addressed the previous reviewers comments well.
- I would like to see a reference to the "Several DSS of different design", Page 3 of 19.
- In recommendations they may wish to add that having communication specialists and health policy people as part of the specialist development team may assist in scaling up and fine tuning to tool for maximum utility. 
- Page 7 under Use Cases remove TO in the sentence used to the outputs from....
- The discussion should also clarify if other than researchers who have used the tool, do they have evaluation data from other target audiences for whom the tool was designed (Table 2) about its utility and user friendliness. Robust qualitative feedback would be useful for others who may wish to use and for the designers' next phase.
- The WHO 2018 guideline on digital health provide some useful approaches to evaluating utility and scale up - albeit of digital apps but could be a framework for this evaluation as well. ( WHO guideline: recommendations on digital interventions for health system strengthening.)

I confirm that I have read this submission and believe that I have an appropriate level of expertise to confirm that it is of an acceptable scientific standard.

# Peer review: Reviewer response for version 1

**DOI:** 10.21956/wellcomeopenres.16096.r35202

**Published:** 2019-04-23

**Authors:** Jon Haël Brenas

**Affiliations:** 1 University of Tennessee Health Science Center, Department of Pediatrics, Oak Ridge National Laboratory, Center for Biomedical Informatics, Memphis, TN, USA

This submission presents an interactive application to display the results of mathematical models in the context of malaria elimination in the Asia-Pacific sphere. This tool aims to address the concerns of a great variety of actors from funding agencies to local staff, to modelers. These different actors have different expectations from the application and different obstacles need to be cleared for them to maximally benefit from it. The submission clearly establishes what these expectations and problems are and presents ways to address them.

 The interactive application seems to be but a part in a massive and timely project and is connected to three more submissions to this publishing platform that present different but complimentary aspects. In my opinion, this does not prevent this submission from having an interesting narrative on its own. That said, the actual description of the application itself is somewhat lacking. Several times in the course of the method section (e.g., "Several modeling scenarios were changed accordingly, leading to alterations in the App", last sentence of subsection "App design"), discussions, presentations and exchanges are mentioned but no detail is given on what were the results. Similarly, target audience's relevant interests and factors limiting their optimal use of the application are listed and some strategies to address them are given but little to nothing is said on how it impacted the development of the application, its distribution or its use. For instance, in subsection "App design", it is explained that the application only displays the pre-computed results of models to save time, which is very a very important decision, but doesn't not discuss how it impacts how users access and use the result and whether it is a problem to limit their ability to fine-tune aspects of the model or not.

 Overall, the evaluation of the application is limited and the actual description of the code created is too short to form an accurate picture of the application development, distribution and use. This submission works very well as a statement of intention to develop the application but not as much as a presentation of its actual development. Most of the discussion section, that I expected to deal with the evaluation of the application, actually explained why such an application is important and not why this specific application solves the problem. To be fair, the submission explains that there is no universally accepted benchmark to evaluate such an application but even a qualitative evaluation is better than no evaluation.

 In my opinion, this submission tried to cast too wide a net and, as a result, fails to answer most of the questions that arise in a satisfactory manner. In my opinion, a much more focused "Use cases" section that would only consider what are the needs of a specific group of users, what are the problems that they encounter and how each one of the five main sections addresses them would make the submission much clearer and provide the missing proof that the application is actually successfully being used.

 Minor remarks:

 1) Table 1 is too prominent, in particular given the fact that it is not used to connect strategies to specific parts of the application .

 2) On the other hand, Figure 1 probably should be bigger to better showcase the actual app.  

 3) A paragraph in section "Discussion" describing what problems are left and ideas on how to solve them would be interesting.

I confirm that I have read this submission and believe that I have an appropriate level of expertise to confirm that it is of an acceptable scientific standard, however I have significant reservations, as outlined above.

# Response

**Published:** 2019-06-27

**Authors:** Richard Maude

**Affiliations:** Mahidol Oxford Tropical Medicine Research Unit, Thailand

We would like to thank the reviewers for their constructive comments and suggestions for the manuscript. We believe that the comments have identified important areas which required improvement. After completion of the suggested edits, the revised manuscript has benefitted from an improvement in the overall presentation and clarity, notably with a much more focused “Use Case” section.

Below, you will find a description of how each comment was addressed in the manuscript. Original reviewer comments in boldface, responses in regular typeface.

We look forward to hearing from you and to respond to any further questions and comments you may have.

**Review #2**

**This submission presents an interactive application to display the results of mathematical models in the context of malaria elimination in the Asia-Pacific sphere. This tool aims to address the concerns of a great variety of actors from funding agencies to local staff, to modelers. These different actors have different expectations from the application and different obstacles need to be cleared for them to maximally benefit from it. The submission clearly establishes what these expectations and problems are and presents ways to address them.**

**The interactive application seems to be but a part in a massive and timely project and is connected to three more submissions to this publishing platform that present different but complimentary aspects. In my opinion, this does not prevent this submission from having an interesting narrative on its own. That said, the actual description of the application itself is somewhat lacking. Several times in the course of the method section (e.g., "Several modeling scenarios were changed accordingly, leading to alterations in the App", last sentence of subsection "App design"), discussions, presentations and exchanges are mentioned but no detail is given on what were the results.**

The building of the App was a non-linear process developed due to an identified need and several discussions with countries and partners that spanned several months. We have added to the *Methods* section two examples of alterations made from the first prototype onwards as the team consulted: the addition of maps to the App and the selection of countries by region.

**Similarly, target audience's relevant interests and factors limiting their optimal use of the application are listed and some strategies to address them are given but little to nothing is said on how it impacted the development of the application, its distribution or its use. For instance, in subsection "App design", it is explained that the application only displays the pre-computed results of models to save time, which is very a very important decision, but doesn't not discuss how it impacts how users access and use the result and whether it is a problem to limit their ability to fine-tune aspects of the model or not.**

The decision to display pre-computed result is indeed very important. We now discuss this decision in the *Methods* section and have added in the *Discussion* on on-going work with an app that allows users to build their own package of interventions.

**Overall, the evaluation of the application is limited and the actual description of the code created is too short to form an accurate picture of the application development, distribution and use. This submission works very well as a statement of intention to develop the application but not as much as a presentation of its actual development. Most of the discussion section, that I expected to deal with the evaluation of the application, actually explained why such an application is important and not why this specific application solves the problem. To be fair, the submission explains that there is no universally accepted benchmark to evaluate such an application but even a qualitative evaluation is better than no evaluation.**

We agree that a formal qualitative evaluation of the App would have provided useful insights. This is a limitation of this manuscript. However, we have informal feedback from country level users and partners including through APLMA and APMEN.

**In my opinion, this submission tried to cast too wide a net and, as a result, fails to answer most of the questions that arise in a satisfactory manner. In my opinion, a much more focused "Use cases" section that would only consider what are the needs of a specific group of users, what are the problems that they encounter and how each one of the five main sections addresses them would make the submission much clearer and provide the missing proof that the application is actually successfully being used.**

We rewrote the Use Case section to explain how the model and App were used by researchers from UCSF to assess the economic rationale of malaria elimination in several national and regional settings. This links the App with the creation of one of five "Elimination investment cases” reports investigating the costs (and economic benefits) of malaria elimination. This provides an example of a practical application of the App. Furthermore, during the dissemination of the Investment cases, demonstrations of the app and the its utility to visualize transmission timelines and costs were found to be extremely useful by the country level users.

**Minor remarks:**

**1) Table 1 is too prominent, in particular given the fact that it is not used to connect strategies to specific parts of the application .**

We shortened Table 1 and cut some elements.

**2) On the other hand, Figure 1 probably should be bigger to better showcase the actual app.**

Figure 1 was replaced with four figures to better showcase the different stages of the App.

**3) A paragraph in section "Discussion" describing what problems are left and ideas on how to solve them would be interesting.**

The discussion section has been expanded to discuss how a country level approach is starting to be used.

# Peer review: Reviewer response for version 1

**DOI:** 10.21956/wellcomeopenres.16096.r35201

**Published:** 2019-04-09

**Authors:** Catherine M. Smith

**Affiliations:** 1 Institute of Health Informatics, University College London, London, UK

This article describes the development of a web-based interactive application to display results of mathematical models for malaria elimination. The app is intended for a range of users including policymakers, staff working in malaria control programs, and researchers. The authors provide a very clear rationale for development of this app, and how other research projects may benefit from development of other similar tools. The app itself is well thought-out, with inclusion of graphics and videos to help users navigate the interface and provide background information. The code is open source and published in an online repository.

 I have only a few suggestions:

- In general, I found that the visualisations produced by the app were easy to use and understand. One exception to this was the “Elimination Timeline” within “Model Stage 2” – I found the “NO” labels confusing when listed alongside the other estimated dates of elimination – is there a reason that the dates could not be shown when they are after 2030? These labels are also a bit redundant for the countries after the 2030 line, which are additionally shown in red. (I would suggest the authors consider adapting this in future versions of the app, although it is not required for this publication).
- In the introduction and discussion, the authors present a strong case for interactive tools like this one as a means of improving communication of complex public health analyses. Since one of the objectives of the app is to engage with a diverse target audience, I would also be interested in how they plan to disseminate the tool to the potential users, and whether they have plans for assessing the impact of the tool. This might include number of hits to the website, questionnaires of users (or people who decided not to use it) or interviews. Evidence of impact would help other researchers assess whether they should invest in creating similar apps.
- Another challenge with this sort of project that is not mentioned is the ongoing support that might be needed to assist users or answer queries. In Table 1, it also says that in case of poor internet connectivity, authors are available to provide support to install an offline version. Could the authors comment on the resources available for support and how long it is expected to be maintained for?

I confirm that I have read this submission and believe that I have an appropriate level of expertise to confirm that it is of an acceptable scientific standard.

# Response

**Published:** 2019-06-27

**Authors:** Richard Maude

**Affiliations:** Mahidol Oxford Tropical Medicine Research Unit, Thailand

We would like to thank the reviewers for their constructive comments and suggestions for the manuscript. We believe that the comments have identified important areas which required improvement. After completion of the suggested edits, the revised manuscript has benefited from an improvement in the overall presentation and clarity, notably with a much more focused “Use Case” section.

Below, you will find a description of how each comment was addressed in the manuscript. Original reviewer comments in boldface, responses in regular typeface.

We look forward to hearing from you and to respond to any further questions and comments you may have.

**Review #1**

**In general, I found that the visualisations produced by the app were easy to use and understand. One exception to this was the “Elimination Timeline” within “Model Stage 2” – I found the “NO” labels confusing when listed alongside the other estimated dates of elimination – is there a reason that the dates could not be shown when they are after 2030? These labels are also a bit redundant for the countries after the 2030 line, which are additionally shown in red. (I would suggest the authors consider adapting this in future versions of the app, although it is not required for this publication).**

The Elimination Timeline graph was updated, removing the labels “NO” for countries that will not achieve elimination by 2030. In the explanatory text provided above the graph, it is now emphasized that “ *’NO means elimination was not predicted to be achieved by 2030 — the last year projected by the model*.” The year 2030 represents the last year for which results are available. The model’s author predicts that going beyond 2030 would create results with too much uncertainty attached to them to be useful.

**In the introduction and discussion, the authors present a strong case for interactive tools like this one as a means of improving communication of complex public health analyses. Since one of the objectives of the app is to engage with a diverse target audience, I would also be interested in how they plan to disseminate the tool to the potential users, and whether they have plans for assessing the impact of the tool.**

We have amended the *Discussion* to include how the app was disseminated. Given that the app was part of a larger project to develop the evidence base for resource mobilization for elimination in the Asia Pacific region, the dissemination of app followed the plan for dissemination of the entire package of evidence. Opportunities for dissemination were identified in collaboration with APLMA and APMEN. These included during "malaria week" In Bangkok in 2017, APMEN annual and working group meetings In 2017 and 2018, partner meetings (for example, Regional Artemisinin Initiative steering committee meetings), WHO meetings and the World Malaria Congress in July 2018. The APMEN meetings included country level malaria programs as well as partner institutions..

**This might include number of hits to the website, questionnaires of users (or people who decided not to use it) or interviews. Evidence of impact would help other researchers assess whether they should invest in creating similar apps.**

We did not plan from the start to monitor the use of the app. We have added to the discussion an explanation on why the application usage metrics collected do not help us form an accurate picture of the app usage. We agree that collecting quantitative or qualitative data from users would have provided useful data on the impact of the App. It is unfortunate that we did not mobilize resources to do so. An assessment plan is already in place for current projects focusing on the application for malaria elimination.

**Another challenge with this sort of project that is not mentioned is the ongoing support that might be needed to assist users or answer queries. In Table 1, it also says that in case of poor internet connectivity, authors are available to provide support to install an offline version. Could the authors comment on the resources available for support and how long it is expected to be maintained for?**

We have added to the *Software Availability* explanations on why the App should remain accessible for the foreseeable future. The grant supporting the project has ended but authors are involved in several similar projects where users will be trained in the use of the applications, and the applications themselves can be updated with new data, ensuring the relevance of App. In other projects, maintenance grants are being sought to sustain support for the applications for an additional period. At the same time, support is available through APMEN and APLMA as this was one of the products developed as part of a larger project and dissemination planned in collaboration and in support of their member countries.
